# New York Odontological Society

**Published:** 1887-12

**Authors:** 


### NEW YORK ODONTOLOGICAL SOCIETY.

REPORTED FOR THE INDEPENDENT PRACTITIONER.

The November meeting of this Society was held on the 8th ult., at the New York Academy of Medicine, Dr. J. Morgan Howe presiding.

Dr. C. E. Francis presented a small syringe for injecting medicine into root-canals, devised by Dr. J. A. Dunn, of Chicago. He said he had used every sort of syringe ever heard of intended for this purpose, but all have proved objectionable. With the ordinary glasstube syringe the piston is apt to stick when pressing the rod, which is likely to bend the tube or cause it to slip, and perhaps get some of the medicine in the mouth or on the lip. Dr. Dunn's syringe is free from such objection, and requires no effort to use. He has never before experienced such satisfaction in treating root-canals as now.

Dr. S. G. Perry described a case of replanting. He also exhibited a matrix made of thin steel ribbon about an inch in length, with a perforation at each end to insert a silk ligature for securing it to the tooth. He claims that the band, when carried between the teeth and fastened, will so bend as to take the original shape of the tooth and enable the operator to pack the filling securely against the cervical margin of the cavity. Dr. Perry had a cast with natural teeth imbedded in the plaster, and a matrix adjusted to show its nice adaptation.

Dr. W. G. A. Bonwill, of Philadelphia, read a lengthy and interesting paper on Amalgam. He gave a history of his experience with this material as a tooth-stopping, with some ideas regarding its preparation, and his method of manipulating it. He spoke of his efforts to combat the prejudices of parties who possess a mortal dread of having any sort of mercurial preparation in their mouths, whether amalgam fillings or rubber plates. Among his patients were several physicians of the Hahnemann school, with each of whom he had held a sort of argumentative combat, and every time silenced their small and poorly arranged batteries. The doctor has much faith in good amalgam fillings, which should be introduced with great thoroughness. An operator who can put in excellent gold fillings is just the one who can use amalgam intelligently. He also favors the use of gutta-percha stoppings, which possess good saving properties. He deprecates the use of gold for the teeth of young children, and uses amalgam or gutta-percha in such cases. He considers tooth-contact a menace of decay, and likes separations between the teeth. He does not believe in using separators to make space for filling proximal cavities. At the close of his reading the doctor demonstrated his manner of mixing and packing his alloy, using much pressure and working the free mercury to the surface by packing against it bits of folded bibulous paper.

Dr. Francis was much interested in the paper of Dr. Bonwill, and particularly in his antagonism with prejudice. He also had met with somewhat similar experiences, and related an instance where a physician once charged his lack of success in treating a patient to mercurial fillings in her teeth, when not a particle of amalgam was present. He did not object to amalgam for fear of any mercurial effect it could have upon the system. This idea of mercurial poisoning he considered nonsensical. His only objection to amalgam was that he could not always rely on it. He had seen cases where it had done good service for many years, yet in many instances it gave out in a comparatively brief time. It was apt to recede from the cavity margins, or the cavity margins to waste around the fillings.

Dr. A. H. Brockway thought the same objection might be made to gold, for that is not always reliable. Gold fillings sometimes fail. He thought the success in use of amalgam often resulted from care in using it and the proper preparation of cavities.

Dr. C. F. Ives also believed that success in use of amalgam depended much on its proper manipulation. He would not use any excess of mercury, but have as little as possible in the filling.

Dr. Jackson had used spunk instead of paper for pressing against amalgam fillings.

Dr. J. W. Clowes assured gentlemen that there need be no fear in using plenty of mercury to mass the filling.

Dr. W. H. Dwinelle said that in some instances it seemed advisable to employ amalgam, and he occasionally used it; not from lack of ability to manipulate gold, even in difficult cases, but sometimes it served a better purpose. Some time ago he restored with amalgam a broken molar which already contained a large contour gold filling, and recently he had seen the tooth and found it in excellent condition. He also had met with people stocked with prejudices and groundless fears concerning mercurial fillings. He referred to the action of the old New York associations of dentists, soon after amalgam came into use, in compelling its members to pledge themselves not to use this material under fear of expulsion, and denouncing as empirics all who employed it. That action would not bind the intelligent dentists of the day, because it was believed to be largely the result of ignorance and prejudice. It resulted in a rupture in the Society, and engendered much ill-feeling.

On motion of Dr. Dwinelle, a vote of thanks was tendered by the Society to Dr. Bonwill for his interesting paper.

